# Book Reviews

**Published:** 1902-09

**Authors:** 


### BOOK REVIEWS.

APPLIED SURGICAL ANATOMY REGIONALLY PRESENTED. For the use of Students and Practitioners of Medicine. By George B. Woolsey, A.B., M.D., New York. Lea Bros. & Co., New York and Philadelphia, 1902.

The object of this book is to throw into more direct relationship the allied sciences of anatomy and surgery. It is written by one who, from long experience in both of these provinces, is admirably equipped to perform the task well. The book is thoroughly comprehensive and fully illustrated, with illustrations mostly in colors. The book will be a valuable addition to the literature of the subject, which is not overcrowded.

A PHYSICIAN'S PRACTICAL GYNECOLOGY. By W. O. Henry, M.D., Omaha, Nebraska. The Review Press, Lincoln, Neb., 1902.

This small, neat work of two hundred odd pages is intended as a practical guide to the study and practice of diseases of women. It is brief, concise, and the text is profusely illustrated. As it is intended merely as a working guide, it is probably sufficiently comprehensive to serve this purpose, and as such will doubtless be found of service.

